# Hyperthermia-induced changes in leukocyte survival and phagocytosis: a comparative study in bovine and buffalo leukocytes

**DOI:** 10.3389/fvets.2023.1327148

**Published:** 2024-01-23

**Authors:** Maria Carmela Scatà, Mohanned Naif Alhussien, Francesco Grandoni, Anna Reale, Michele Zampieri, Jamal Hussen, Giovanna De Matteis

**Affiliations:** ^1^Research Centre for Animal Production and Aquaculture, Council for Agricultural Research and Economics (CREA), Rome, Italy; ^2^Reproductive Biotechnology, TUM School of Life Sciences, Technical University of Munich, Freising, Germany; ^3^Department of Experimental Medicine, Faculty of Medicine and Dentistry, Sapienza University of Rome, Rome, Italy; ^4^Department of Microbiology, College of Veterinary Medicine, King Faisal University, Al-Ahsa, Saudi Arabia

**Keywords:** heat stress, leukocytes, apoptosis, phagocytosis, bovine, buffalo, flow cytometry

## Abstract

Heat stress negatively affects health, welfare, and livestock productivity by impairing immune function, increasing disease incidence. In recent years, there has been increasing interest in understanding the immune system of water buffalo due to the growing economic impact of this species for the high quality and nutritional value of buffalo milk. While there are common responses across bovine and buffalo species, there are also some species-specific variations in the physiological responses to heat stress, mainly attributed to differences in metabolism and heat dissipation efficiency. At cellular level, the exposure to thermal stress induces several anomalies in cell functions. However, there is limited knowledge about the differential response of bovine and buffalo leucocytes to early and late exposure to different degrees of thermal exposure. The aim of this study was to compare the *in vitro* effect of hyperthermia on apoptosis and phagocytosis in leukocytes from bovine and buffalo species. For this, whole blood samples of six bovines and nine buffaloes were incubated at 39°C (mimicking normothermia condition) or 41°C (mimicking heat stress condition) for 1, 2, and 4 h. Two flow cytometric assays were then performed to evaluate apoptosis and determine functional capacity of phagocytic cells (neutrophils and monocytes). The results showed that the viability of bovine and buffalo leukocytes was differently affected by temperature and time of *in vitro* exposure. A higher percentage of apoptotic leukocytes was observed in bovines than in buffaloes at 39°C (3.19 vs. 1.51, *p* < 0.05) and 41°C (4.01 vs. 1.69, *p* < 0.05) and for all incubation time points (*p* < 0.05). In contrast, no difference was observed in the fraction of necrotic leukocytes between the two species. In both species, lymphocytes showed the highest sensitivity to hyperthermia, showing an increased apoptosis rates along with increased incubation time. In bovine, apoptotic lymphocytes increased from 5.79 to 12.7% at 39°C (*p* < 0.05), in buffalo, this population increased from 1.50 to 3.57% at 39°C and from 2.90 to 4.99% at 41°C (*p* < 0.05). Although no significant differences were found between the two species regarding the percentage of phagocytic neutrophils, lower phagocytosis capacity values (MFI, mean fluorescence intensity) were found in bovines compared with buffaloes at 41°C (27960.72 vs. 53676.45, *p* > 0.05). However, for monocytes, the differences between species were significant for both phagocytosis activity and capacity with lower percentages of bovine phagocytic monocytes after 2 h at 39°C and after 1 h at 41°C. The bovine monocytes showed lower MFI values for all temperature and time variations than buffaloes (37538.91 vs. 90445.47 at 39°C and 33752.91 vs. 70278.79 at 41°C, *p* < 0.05). In conclusion, the current study represents the first report on the comparative analysis of the effect of *in vitro* heat stress on bovine and buffalo leukocyte populations, highlighting that the leukocytes of buffalo exhibit relatively higher thermal adaptation than bovine cells.

## Introduction

In the last years, much research has been carried out to study heat stress in livestock and investigate the effects of increased global temperatures on the health and welfare of farm animals. Based on mathematical models, the global average temperature would rise to an estimated 3.28°C (2.46–4.10°C) above its pre-industrial level by 2,100 (1). Several studies have shown that high environmental temperature negatively affects health and biological functions of dairy cows, inducing reduced milk production and reproductive performances (2–5). Recent studies have focused on the impact of heat stress on the immune response in farm animals (6–9). Lemal et al. (2) reviewed the effect of heat stress on diseases and immune response in cattle, highlighting that heat stress triggers the hypothalamic–pituitary–adrenal axis which stimulate cortisol secretion in the bloodstream. The target cells of cortisol are the immune cells. The complex formed by cortisol and its receptor reduces the ability of immune cells to react to infection and limits the recruitment of immune cells to the site of infection. Furthermore, cortisol has been found to hamper the expression of pro-inflammatory cytokines as TNF-α, a cytokine involved in cellular proliferation and apoptosis. Lendez et al. (3) showed that under heat stress conditions, the expression of TNF-α and its receptor decreases in dairy cows.

*In vitro* heat stress conditions caused decreased viability and increased apoptotic rate in bovine monocytes (10). Changes in leukocyte cell viability and function have also been reported, including reduced phagocytosis and respiratory burst (production of reactive oxygen species) in bovine polymorphonuclear cells (11). Apoptotic cell death is an evolutionary conserved and genetically programmed process of multi-cellular organisms that normally occurs during development and in aged cells. Caspase activation is a well-known key event in the apoptotic process, and caspase-3 activity is the common effector of most of the apoptotic pathways. There are a wide variety of stimuli and conditions, both physiological and pathological, which can trigger apoptosis. Heat stress is an external stimulus that can cause cell damage and is one of the important apoptosis-inducing stresses (11). Wohlgemuth et al. (12) reported that high temperature reduces the proliferation activity of bovine mammary epithelial cells and induces apoptosis. Recently, a model to explore the biological adaptation of the immune system to high temperatures was proposed in dromedary camel, where the *in vitro* exposure of whole blood to hyperthermia leads to an increase of phagocytosis and ROS production in phagocytes (13). Phagocytosis represents the first line of host defense against pathogens and is a central component of innate immunity.

Functional analysis of immune cells is widely performed using flow cytometry, which can also be used to monitor apoptosis and phagocytosis. Flow cytometry is a versatile tool that can benefit various disciplines. It is mostly used for immunophenotyping, the identification and quantification of immune cells and their activity levels in mixed samples, such as blood to examine cellular processes.

In recent years, there has been increasing interest in understanding the immune system of water buffalo (*Bubalus bubalis*) due to the growing economic impact of this species for the high quality and nutritional value of buffalo milk, which is particularly suitable for manufacturing of fresh cheese (14). While buffaloes are naturally adapted to heat stress, they show signs of severe distress when exposed to direct solar radiation. This vulnerability stems from having darker, thicker skin and fewer sweat glands than cattle. These distinct characteristics of buffaloes hinder effective heat dissipation and render them more susceptible to heat stress (15). Bagath et al. (8) have exhaustively described the impact of heat stress on the immune system in dairy cattle, concluding that acute heat stress may have a stimulatory effect on the immune system. In contrast, chronic heat stress seems to have an inhibitory role on the capacity of the immune system to maintain homeostasis.

There is limited knowledge about the differential response of bovine and buffalo leucocytes to early and late exposure to different degrees of heat shock. This knowledge is the key to discovering differences between cows and buffaloes in the immune response to hyperthermia by bringing to light differences in thermotolerance and thus susceptibility to heat stress. Furthermore, this understanding can pave the way for developing strategies to mitigate the adverse effects of heat stress on these animals, optimizing their health and productivity. Consequently, the current study was undertaken to investigate the impact of *in vitro* hyperthermia on the immune cells functions in bovine and buffalo species. To accomplish this, we employed two flow cytometric assays to assess alterations in apoptosis and phagocytosis of bovine and buffalo leukocytes exposed to temperatures of 39 and 41°C for 1, 2, and 4 h. These allowed us to evaluate the effects of short- and long-term heat shock and compare thermotolerance of these two species.

## Materials and methods

### Animals and blood sampling

The study was planned and performed at the CREA Research Center for Animal Production and Aquaculture experimental farm, which was authorized to use farm animals for experimental design (as stated in DM 26/96–4 of the Italian Health and Welfare Ministry). The management and care of the animals were carried out in compliance with the 2010/63/EU directive and the Italian regulation D. Lgs n. 26/2014. The trial was carried out from March to June when animals were not exposed to heat stress conditions. Meteorological records from the city of Monterotondo (Italy)^1^ were used to collect data on temperature and humidity during blood sampling. The average monthly temperature ranged from a minimum temperature of 12.05°C to a maximum temperature of 22.52°C. The average monthly humidity for the same period ranged between 56.49 and 53.56%.

Blood was collected from multiparous lactating cows (from 2 to 5 lactations), 6 Holstein, and 9 Mediterranean buffalo. Before blood sampling, cows were subjected to routine veterinary examinations to exclude animals with clinical signs and symptoms of diseases. Blood was obtained by venipuncture of the external jugular vein into lithium heparin vacutainer tubes (BD Biosciences, San Jose, CA, United States).

### Experimental design

**Figure 1:**
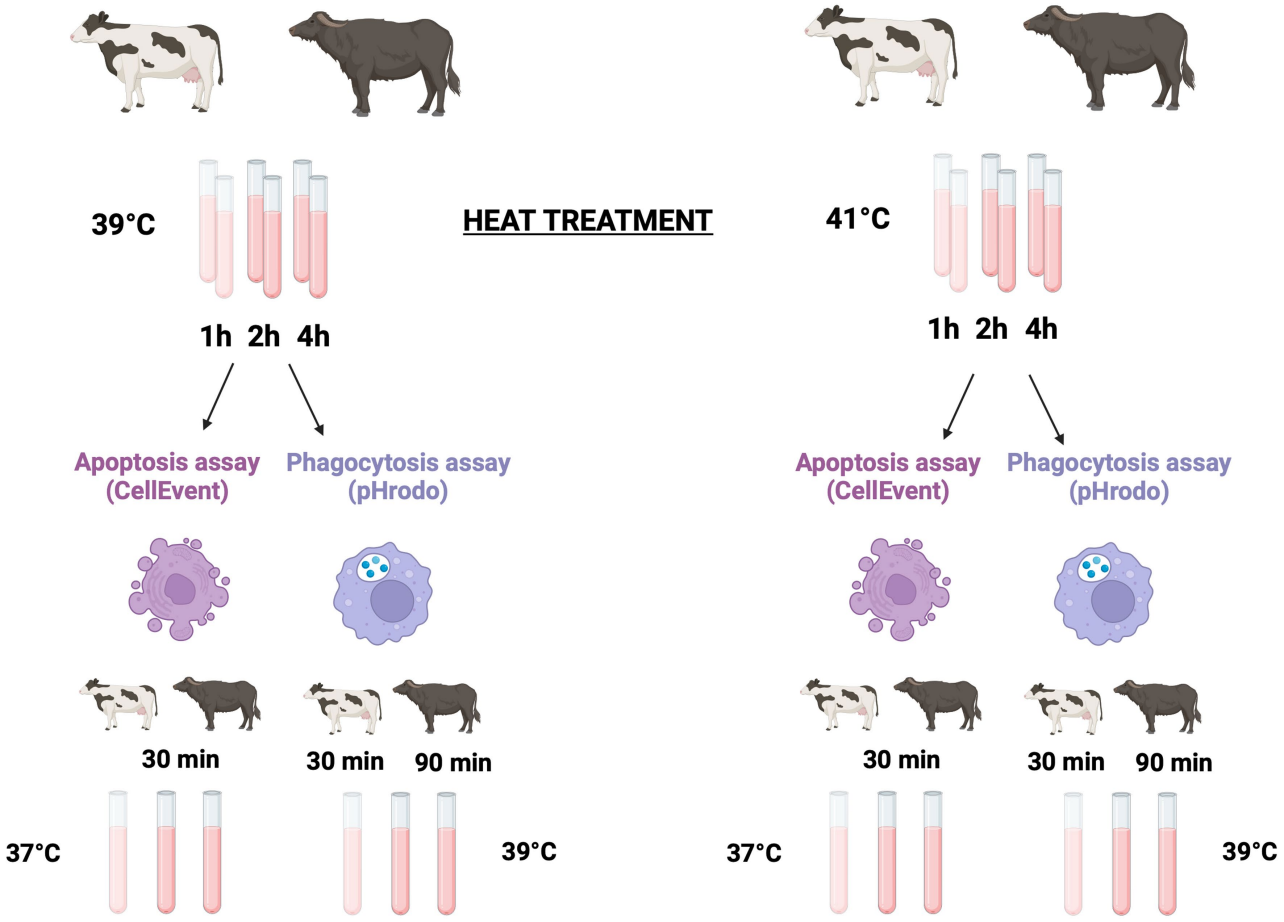
Experimental design. Six Holstein and nine Mediterranean buffalo were used to explore the effect of hyperthermia on apoptosis and phagocytosis. Six experimental tubes containing 100 μL of whole blood from each animal were incubated in a thermostatic water bath either at 39°C (mimicking normothermia condition) and at 41°C (mimicking severe heat stress condition) for 1, 2, and 4 h, respectively. To detect apoptosis and cell death, after the heat treatment, samples were kept on ice for 10 min and then lysed and washed. Leukocytes were incubated for 30 min at 37°C in the presence of the CellEvent Caspase-3/7 green assay. After that, cells were immediately collected in a flow cytometer. To analysis the phagocytosis, after the heat treatment, all tubes were kept on ice for 10 min. Then, pHrodo™ Green *E. coli* Bioparticles^®^ were added to each tube. To start phagocytosis, samples were incubated at 39°C. Bovine samples were incubated for 30 min while buffalo samples were incubated for 90 min. After incubation, phagocytosis was stopped by placing the samples on ice for 10 min, and erythrocytes were then lysed and washed before the flow cytometric analysis. Created with BioRender.com.

To explore the effect of hyperthermia on cell viability, apoptosis, and phagocytosis, we performed an *in vitro* temperature treatment of blood samples. Six experimental tubes containing 100 μL of whole blood from each animal were incubated in a thermostatic water bath at 39°C (mimicking normothermia condition) and 41°C (mimicking severe heat stress condition) for 1, 2, and 4 h. The flowchart of operative procedures is shown in Figure 1.

### Apoptosis assay

The CellEvent Caspase-3/7 Green Assay Kit (Thermo Fisher Scientific, Waltham, MA, United States) was used to assess the caspase activation by flow cytometry, a feature of the early stages of apoptosis. This reagent, a 4 amino acid peptide (DEVD) conjugated to a nucleic acid-binding dye, detects activated caspases-3 and 7 in apoptotic cells. During apoptosis, this sequence is recognized and cleaved by the activated enzymes. The cleavage of the recognition sequence and the binding of DNA by the dye allow to label apoptotic cells. In this kit, the CellEvent Caspase-3/7 is used together with the SYTOX AADvanced Dead Cell Stain, enabling straightforward differentiation between live, apoptotic, and necrotic cells. After the heat treatment, samples were lysed with 1 mL of Tris-buffered ammonium chloride solution (0.87% w/v, pH 7.3) at room temperature for 10 min. Lysis was stopped with 3 mL of cold PBS, and leukocytes were recovered by centrifugation at 300 × g for 5 min at 4°C. Cells were resuspended in 1 mL of PBS/2% BSA and incubated for 30 min at 37°C in a humidified CO_2_ incubator in the presence of 1 mL of the CellEvent Caspase-3/7 Green Detection Reagent. During the final 5 min of staining, 1 mL of the 1 mM SYTOX AADvanced Dead Cell Stain was added to the samples. Samples were then immediately acquired on a CytoFLEX flow cytometer (Beckman Coulter, Brea, CA, United States). Each reagent was excited with a 488-nm laser, and the fluorescence emission was collected using a 525/40 bandpass filter for CellEvent Caspase-3/7 Green Detection Reagent and a 690/50 bandpass filter for SYTOX AADvanced Dead Cell Stain. Single stained sample controls of both reagents were also analyzed to adjust at the compensation level.

### Phagocytosis assay

The pHrodo™ Green *E. coli* Bioparticles^®^ Conjugated (Thermo Fisher Scientific, Waltham, MA, United States) was used to assess phagocytosis in whole blood samples by flow cytometry. After the heat treatment, all experimental tubes were kept on ice for 10 min. Then, 20 μL of 1 mg/mL of pHrodo™ Green *E. coli* Bioparticles^®^ were added to each tube. To start phagocytosis, samples were incubated at 39°C.

To test different incubation times (30, 60, 90, and 120 min) and pHrodo™ Green *E. coli* Bioparticles^®^ cell ratio (5:1; 10:1; and 20:1) for bovine and buffalo species, preliminary experiments were carried out. Based on operational consideration and the higher level of phagocytosis found in both species (data not shown), we identified the incubation time of 30 and 90 min for bovine and buffalo samples, respectively.

For each experimental condition, one tube was maintained on ice as a negative control sample (phagocytosis inhibition). After incubation, phagocytosis was stopped by placing the samples on ice for 10 min. Erythrocytes were then lysed with 1 mL of Tris-buffered ammonium chloride solution (0.87% w/v, pH 7.3) at room temperature for 10 min. All tubes were centrifugated at 300 × g for 5 min at 4°C and washed twice with PBS. The pHrodo™ dye is highly fluorescent in the acidic environment of the phagosome upon internalization, and then, this property eliminates the quenching step. Cells were resuspended in 100 μL of PBS and immediately collected on a CytoFLEX flow cytometer. The pHrodo™ Green *E. coli* Bioparticles^®^ were excited with a 488-nm laser using a 525/40 bandpass filter. Kaluza Analysis software v 2.1 (Beckman Coulter, Brea, CA, United States) was used to analyze the flow cytometric data. To analyze samples, we set up dot plots showing forward scatter (FSC) vs. side scatter (SSC) to select granulocytes and monocytes and a dot plot showing SSC vs. fluorescence to measure the fluorescence. Neutrophils and eosinophils were easily separated by the increased autofluorescence of the latter population in all fluorescent channels. The percentages of phagocytic cells as neutrophils and monocytes (phagocytic activity) were analyzed, and their MFI is the expression of the number of bacteria ingested (phagocytic capacity).

### Statistical analysis

Data were analyzed using SPSS software (IBM, version 22), and graphs were created using GraphPad Prism (version 8.3.1). Pairwise comparisons were conducted using a multiple comparison test (Tukey’s test). All data were expressed as mean ± S.E and analyzed with repeated measures three-way ANOVA test. Mean values were tested for significance at 5 and 1% levels. The following statistical model was employed to assess the impact of species, temperature, duration of heat stress exposure, and their interactions on the dependent variable:


$$\begin{array}{l} Y_{ijk}=\mu +S_{i}+T_{j}+D_{k}+{\left(ST\right)}_{ij}+{\left(SD\right)}_{ik}\\ \;+{\left(TD\right)}_{jk}+{\left(STD\right)}_{ijk}+e_{ijk} \end{array}$$

In this model, Y_ijk_ represents the dependent variable, μ is the overall population mean, S_i_ represents the species effect (i = 2 for two species), T_j_ represents the temperature effect (j = 2 for two temperature levels), D_k_ represents the heat stress duration effect (k = 3 for three duration levels), and e_ijk_ represents the random error, assumed to be independent and normally distributed.

## Results

### Effect of hyperthermia on bovine and buffalo leukocytes viability

**Figure 2:**
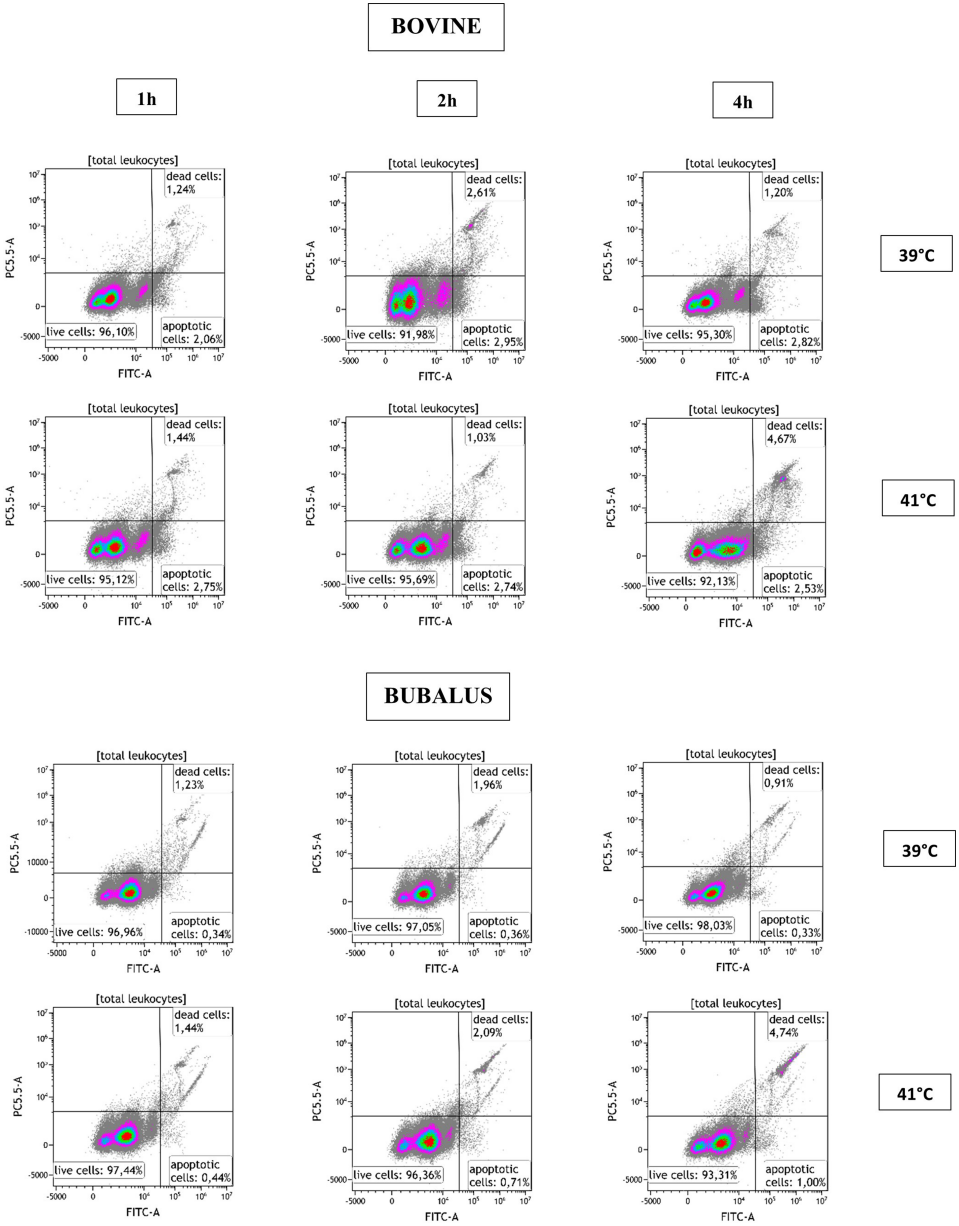
Flow cytometric analysis of apoptosis with activation of Caspase-3/7. After the exclusion of cell doublets, the leukocytes were identified based on their FSC and SSC characteristics and were plotted in a dual parameter dot plot CellEvent Caspase-3/7 (FITC-A) vs. SYTOX AADvanced Dead Cell fluorescence. Three populations were identified (live, necrotic, and apoptotic cells) and calculated for blood sample after incubation at 39 and 41°C for 1, 2, and 4 h.

To evaluate the effect of temperature on the viability of cells, we used a flow cytometric assay that allows to measure early stage of apoptosis and late cell necrosis at the same time. As shown in Figure 2, in a dual parameter dot plot of CellEvent Caspase-3/7 vs. SYTOX AADvanced Dead Cell fluorescence, three cell populations can be distinguished (live, necrotic, and apoptotic cells), thereby revealing the effect of hyperthermia on the viability of leukocytes.

Table 1 shows the mean percentages of apoptotic and necrotic leukocytes, granulocytes, monocytes, and lymphocytes, after incubation at 39 or 41°C for 1, 2, and 4 h.

**Table 1:** Comparison of the percentage values of apoptotic and necrotic leukocyte subsets between bovine and buffalo after *in vitro* incubation at 39°C (normal temperature) and 41°C (hyperthermia).

	Bovine		Buffalo		*p-*value						
Temperature/subset	39°C	41°C	39°C	41°C	Species	Temperature	Time	Species * temperature	Species * time	Temperature * time	Species * temperature * time
Apoptotic leukocytes (%)	3.19 ± 0.37^**B**^	4.01 ± 0.54^**B**^	1.51 ± 0.17^**A**^	1.69 ± 0.16^**A**^	**≤0.001**	0.136	0.155	0.339	0.725	0.500	0.907
Necrotic leukocytes (%)	4.61 ± 0.98	7.57 ± 1.84	5.23 ± 0.78	6.96 ± 1.12	0.978	0.055	**0.028**	0.623	0.337	0.435	0.679
Apoptotic granulocytes (%)	1.99 ± 0.31	2.08 ± 0.37	2.19 ± 0.34	2.07 ± 0.35	0.784	0.965	0.819	0.823	0.931	0.407	0.414
Necrotic granulocytes (%)	1.49 ± 0.29^**A**^	2.54 ± 0.50^**B**^	0.97 ± 0.14^**A**^	1.22 ± 0.18^**A**^	**0.001**	**0.021**	0.312	0.172	**0.006**	0.072	**0.039**
Apoptotic monocytes (%)	4.27 ± 0.71^**A**^	7.8 ± 1.09^**B**^	3.58 ± 0.36^**A**^	4.05 ± 0.66^**A**^	**0.003**	**0.007**	0.616	**0.032**	0.53	0.848	0.373
Necrotic monocytes (%)	0.96 ± 0.33	1.13 ± 0.30	0.82 ± 0.13	1.03 ± 0.25	0.596	0.450	**0.005**	0.919	0.255	0.662	0.785
Apoptotic lymphocytes (%)	8.63 ± 1.41^**B**^	0.99 ± 1.74^**B**^	2.32 ± 0.28^**A**^	3.74 ± 0.42^**A**^	**≤0.001**	0.059	**0.018**	0.624	0.582	0.537	0.534
Necrotic lymphocytes (%)	1.05 ± 0.42^**AB**^	2.89 ± 1.17^**C**^	0.85 ± 0.09^**A**^	1.43 ± 0.23^**BC**^	0.114	**0.018**	**≤0.001**	0.220	**0.011**	**0.045**	0.271

Means with different capital superscripts (A, B, and C) differ significantly (*p* < 0.05) in column (39°C vs. 41°C and Bovine vs. Buffalo). Data are presented as means ± SE. Significant values are shown in bold.

Furthermore, the effects of species, temperature, time, and interactions between the different variables are presented. In bovine cows, a significant increase in necrotic lymphocytes and granulocytes and apoptotic monocytes was observed after incubation at 41°C compared with 39°C. In buffaloes, the incubation at 41°C caused an increase only in necrotic lymphocytes (*p* < 0.05). Overall, higher values in bovines were observed for the fraction of apoptotic leukocytes (*p* ≤ 0.001), necrotic granulocytes (*p* = 0.001), apoptotic monocytes (*p* = 0.003), and apoptotic lymphocytes (*p* ≤ 0.001) compared with buffaloes. After incubation at 41°C of bovine cells, we observed a significant increase in necrotic granulocytes (*p* = 0.021), apoptotic monocytes (*p* = 0.007), and necrotic lymphocytes (*p* = 0.018). The effect of incubation time was observed on necrotic leukocytes (*p* = 0.028), monocytes (*p* = 0.005), and apoptotic and necrotic lymphocytes (*p* = 0.018 and *p* ≤ 0.001, respectively). An interaction species*temperature was observed in apoptotic monocytes (*p* = 0.032) while an interaction species*time influenced the percentage of necrotic granulocytes (*p* = 0.006) and lymphocytes (*p* = 0.011); this last population was affected by interaction temperature*time (*p* = 0.045). Finally, the interaction of all factors (species*temperature*time) significantly affected the percentage of necrotic granulocytes (*p* = 0.039).

**Figure 3:**
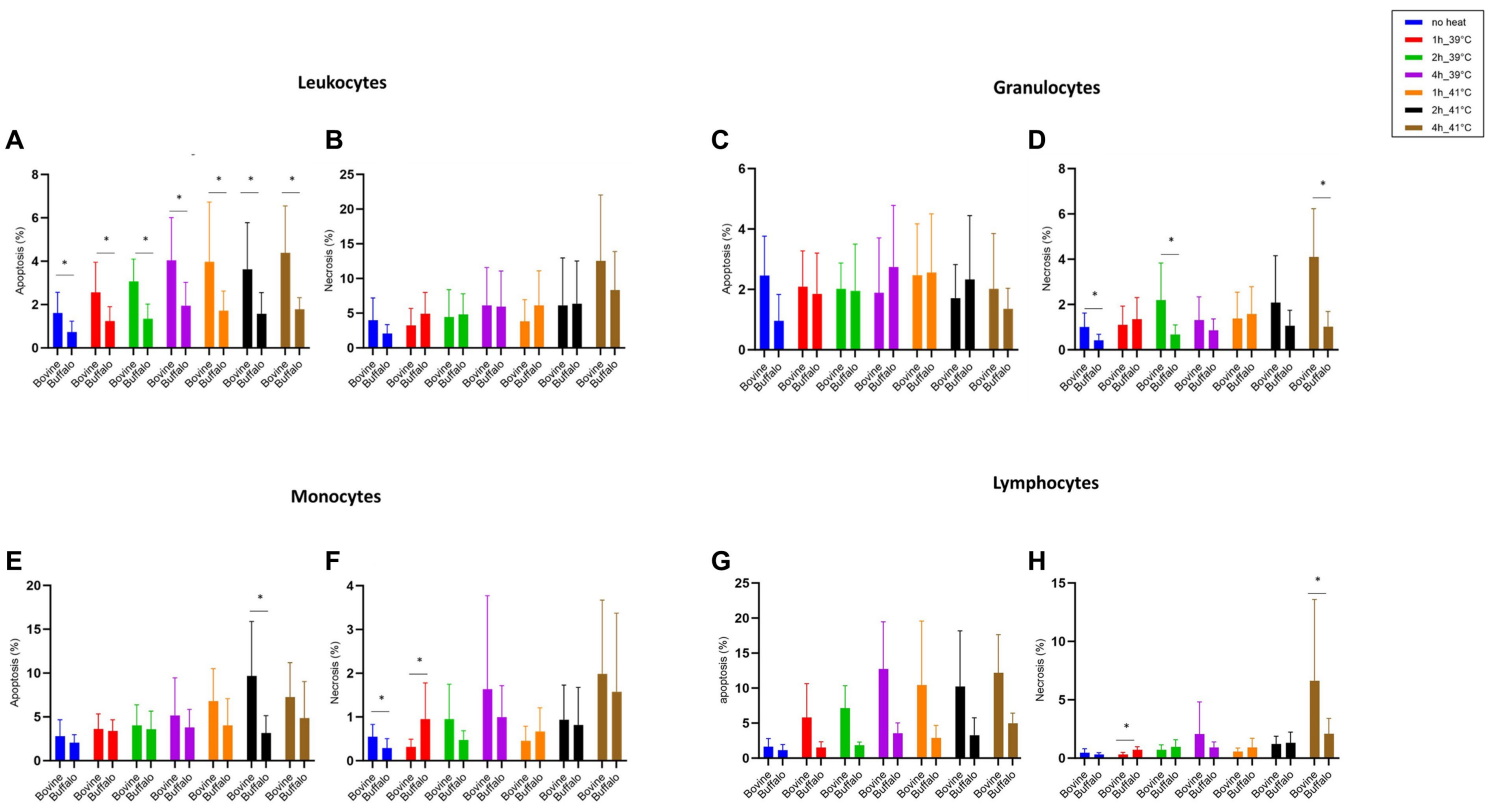
The impact of hyperthermia on leukocytes cell apoptosis and necrosis. The blood samples were incubated at different times (1, 2, and 4 h) and temperatures (39 and 41°C). For both species, no heat sample was used as control for basal level. The percentages of apoptotic and necrotic cells were calculated for gated total leukocytes, granulocytes, monocytes, and lymphocytes and presented as mean ± SEM (*indicates *p* < 0.05).

Figure 3 shows how the cell viability of bovine and buffalo leukocytes was differently affected by hyperthermia and time of incubation. A higher proportion of apoptotic leukocytes was observed in bovine than buffaloes at both temperatures (39 and 41°C) and for all time points (Figure 3A), while no difference was observed in the percentage of necrotic leukocytes between the two species (Figure 3B). Although the fraction of apoptotic granulocytes did not differ significantly between the two species (Figure 3C), a significant increase in necrotic granulocytes was found after 4 h of incubation of bovine cells at 41°C compared with buffaloes (Figure 3D).

For monocytes, cell apoptosis was higher in bovines than buffaloes after 2 h of incubation at 41°C (Figure 3E). However, no difference was found in monocyte necrosis (Figure 3F). Apoptotic lymphocytes showed a pattern which is similar to that of total leukocytes, although the differences between bovine and buffaloes were not significant (Figure 3G). In contrast, necrotic lymphocytes were significantly higher after 1 h at 39°C in buffaloes compared with bovine, while higher values were found in bovine compared with buffaloes after 4 h at 41°C (Figure 3H).

The impact of the time during the *in vitro* heat stress exposure (1, 2, and 4 h) on cell viability of leukocytes and their subpopulations is presented in Table 2. In the bovines, the incubation of blood at 39°C caused an increase in apoptotic leukocytes with significantly higher values after 2 and 4 h compared with baseline values before incubation. However, an increase in apoptotic cells was observed after 1 h when the blood was incubated at 41°C. No increase of necrotic leukocytes was observed during the time course at 39°C, while incubation at 41°C increased necrotic leukocytes after 2 and 4 h compared with baseline values before incubation. A similar pro-necrotic effect was observed in granulocytes from blood samples incubated for 4 h at 41°C compared with 39°C. A substantial increase in apoptotic monocytes was observed early after incubation at 41°C with a significant increase after 1 h of incubation and a maximum increase after 2 h. The lymphocyte population showed the highest increase in cell apoptosis with increased incubation time compared with monocytes and granulocytes. This increase was found after 4 h for samples incubated at 39°C and after 1, 2, and 4 h for those samples incubated at 41°C.

**Table 2:** Comparison of apoptotic and necrotic cells in bovine and buffalo animals at different times and temperatures.

	Apoptotic leukocytes (%)		Necrotic leukocytes (%)		Apoptotic granulocytes (%)		Necrotic granulocytes (%)		Apoptotic monocytes (%)		Necrotic monocytes (%)		Apoptotic lymphocytes (%)		Necrotic lymphocytes (%)	
Bovine																
No heat	1.60 ± 0.42^a^	1.60 ± 0.42^a^	3.96 ± 1.44	3.96 ± 1.44^a^	2.46 ± 0.58	2.46 ± 0.58	1.00 ± 0.27^a^	1.00 ± 0.27^a^	2.79 ± 0.84	2.79 ± 0.84^a^	0.54 ± 0.12^ab^	0.54 ± 0.12^a^	1.63 ± 0.52^a^	1.63 ± 0.52^a^	0.45 ± 0.15^ab^	0.45 ± 0.15^a^
Temperature	**39°C**	**41°C**	**39°C**	**41°C**	**39°C**	**41°C**	**39°C**	**41°C**	**39°C**	**41°C**	**39°C**	**41°C**	**39°C**	**41°C**	**39°C**	**41°C**
1 h	2.47 ± 0.51^ab^	3.97 ± 1.12^b^	3.22 ± 1.00	3.82 ± 1.27^a^	2.09 ± 0.48	2.46 ± 0.69	1.09 ± 0.34^ab^	1.37 ± 0.47^a^	3.60 ± 0.70^**A**^	6.78 ± 1.51^b**B**^	0.31 ± 0.07^a^	0.46 ± 0.13^a^	5.79 ± 1.97^ab^	10.42 ± 3.73^b^	0.32 ± 0.06^a^	0.56 ± 0.12^a^
2 h	3.07 ± 0.45^b^	3.63 ± 0.95^b^	4.45 ± 1.76	6.13 ± 3.05^ab^	2.02 ± 0.38	1.71 ± 0.49	2.19 ± 0.72^b^	2.08 ± 0.92^a^	4.01 ± 1.05^**A**^	9.66 ± 2.78^b**B**^	0.94 ± 0.35^b^	0.94 ± 0.35^ab^	7.13 ± 1.43^ab^	10.24 ± 3.54^b^	0.71 ± 0.19^ab^	1.21 ± 0.29^b^
4 h	4.03 ± 0.80^bc^	4.38 ± 0.88^b^	6.12 ± 2.21	12.5 ± 3.87^b^	1.88 ± 0.74	2.02 ± 0.74	1.31 ± 0.41^ab**A**^	4.1 ± 0.86^b**B**^	5.17 ± 1.75	7.26 ± 1.60^b^	1.63 ± 0.87^b^	1.98 ± 0.68^b^	12.7 ± 2.75^b^	12.18 ± 2.22^b^	2.06 ± 1.12^b**A**^	6.63 ± 2.83^c**B**^
Buffalo																
No heat	0.73 ± 0.20^a^	0.73 ± 0.20^a^	2.07 ± 0.51^a^	2.07 ± 0.51^a^	0.95 ± 0.35^a^	0.95 ± 0.35^a^	0.42 ± 0.10^a^	0.42 ± 0.10^a^	2.03 ± 0.37^a^	2.03 ± 0.37^a^	0.28 ± 0.08^a^	0.28 ± 0.08^a^	1.13 ± 0.33^a^	1.13 ± 0.33^a^	0.30 ± 0.06^a^	0.30 ± 0.06^a^
Temperature	**39°C**	**41°C**	**39°C**	**41°C**	**39°C**	**41°C**	**39°C**	**41°C**	**39°C**	**41°C**	**39°C**	**41°C**	**39°C**	**41°C**	**39°C**	**41°C**
1 h	1.23 ± 0.23^ab^	1.71 ± 0.32^b^	4.88 ± 1.10^b^	6.11 ± 1.75^b^	1.85 ± 0.47^ab^	2.56 ± 0.68^b^	1.35 ± 0.33^b^	1.57 ± 0.42^b^	3.38 ± 0.45^b^	4.03 ± 1.07^b^	0.95 ± 0.29^b^	0.66 ± 0.19^ab^	1.50 ± 0.29^a**A**^	2.90 ± 0.62^b**B**^	0.70 ± 0.09^b^	0.90 ± 0.28^b^
2 h	1.34 ± 0.25^ab^	1.56 ± 0.37^b^	4.82 ± 1.12^b^	6.37 ± 2.32^b^	1.95 ± 0.58^ab^	2.32 ± 0.79^ab^	0.67 ± 0.15^a^	1.06 ± 0.25^b^	3.59 ± 0.78^b^	3.16 ± 0.74^ab^	0.47 ± 0.07^a^	0.81 ± 0.32^ab^	1.83 ± 0.17^a**A**^	3.27 ± 0.94^bc**B**^	0.96 ± 0.23^b^	1.30 ± 0.34^b^
4 h	1.94 ± 0.38^b^	1.77 ± 0.19^b^	5.95 ± 1.80^b^	8.33 ± 1.96^b^	2.74 ± 0.72^b^	1.36 ± 0.23^a^	0.86 ± 0.17^ab^	1.02 ± 0.23^b^	3.79 ± 0.73^b^	4.86 ± 1.47^b^	0.99 ± 0.25^b^	1.57 ± 0.63^b^	3.57 ± 0.51^b**A**^	4.99 ± 0.50^c**B**^	0.90 ± 0.17^b**A**^	2.08 ± 0.46^c**B**^

Means with different lowercases (a, b, and c) differ significantly (*p <* 0.05) in row (no heat vs. 1, 2, or 4 h); means with different capital superscripts (A, B, and C) differ significantly (*p <* 0.05) in column (39°C vs. 41°C). Data are presented as means ± SE.

In the total leukocyte and the granulocyte population of buffalo, the incubation at 39°C caused an increase in apoptotic cells with significantly higher values after 4 h. In contrast, blood exposure at 41°C caused significant increase in cell apoptosis after 1 h compared with baseline values before heat treatment. Incubation at 41°C resulted in a significant increase in necrotic granulocytes at all time points. For monocytes, cell apoptosis was induced with increased incubation time at both temperatures, while the increase in cell necrosis was observed after 4 h at both temperatures. Lymphocytes showed a significant increase in cell apoptosis after 4 h of incubation at 39°C and for each time point at 41°C, while the increase in necrotic lymphocytes started after 1 h of incubation at 41°C with a continuous increase until 4 h (Table 2).

### Effect of hyperthermia on phagocytosis activity of bovine and buffalo neutrophils and monocytes

**Figure 4:**
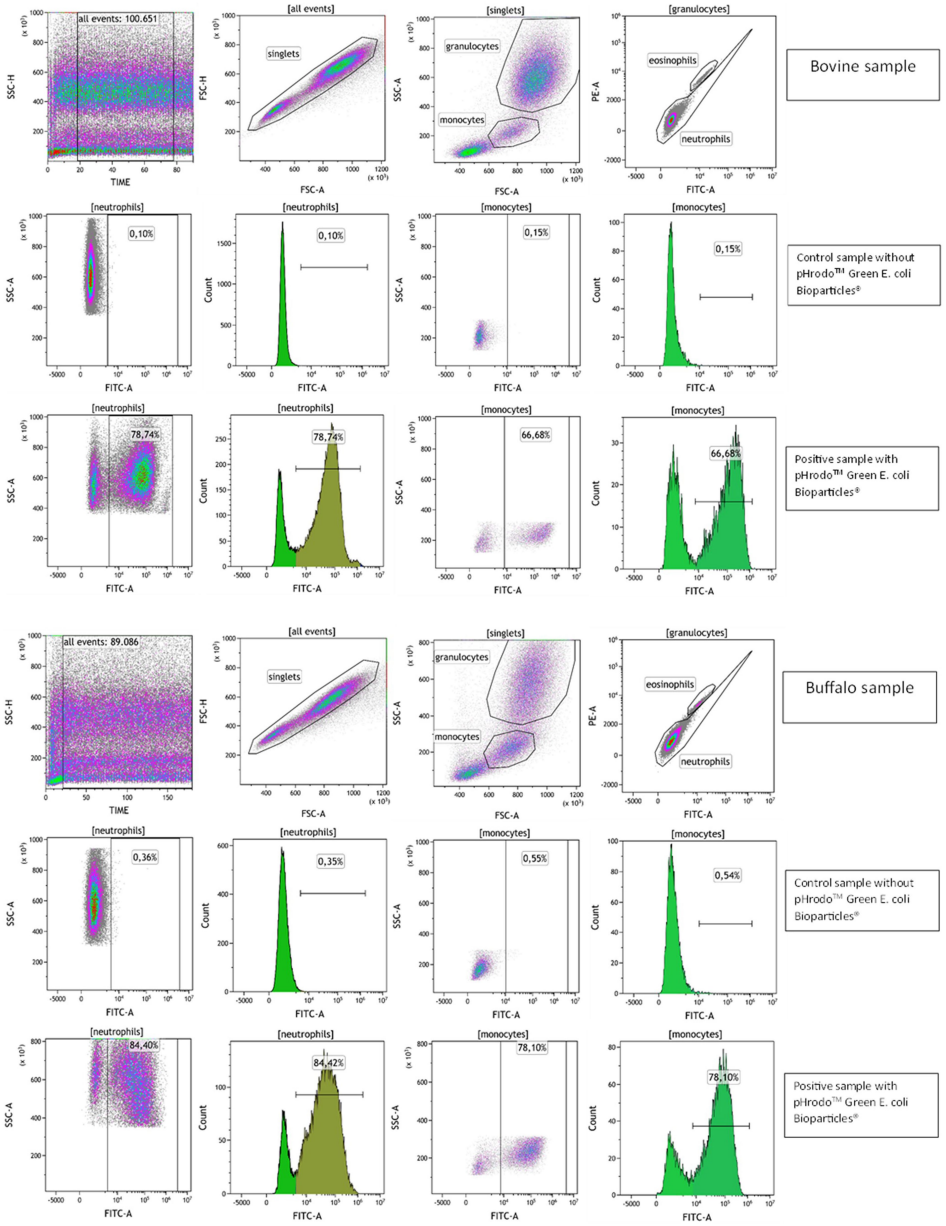
Flow cytometric gating strategy to evaluate phagocytosis in bovine and buffalo blood samples. A time parameter vs. SSC dot plot was used to exclude event burst and a FSC-A vs. FSC-H dot plot to exclude doublets. The granulocytes and monocytes were identified based on their SSC and FSC characteristics, and eosinophils were excluded on dot plot PE vs. FITC channel due to their autofluorescence. The bovine and buffalo blood samples were incubated without (control samples) and with pHrodo™ Green *E. coli* Bioparticles^®^ (positive samples), and the percentages of neutrophils and monocytes pHrodo-positive cells were determined.

Phagocytosis was evaluated by flow cytometry with the pHrodo™ dye-based system that measures phagocytic activity based on acidification of the particles as they are ingested, eliminating the wash and quenching steps. This reagent was previously validated as a rapid and reliable tool for evaluating phagocytosis of multiple species (16). The detector settings of the cytometer were optimized using unlabeled samples. Blood without pHrodo™ Green *E. coli* Bioparticles^®^ was used to set a marker for FL1 autofluorescence so that less than 3% of the events were positive. The percentage of phagocytosing cells (phagocytosis activity) in the experimental sample was determined by identifying the fraction of events above this marker position, while phagocytosis capacity was identified as the mean fluorescence intensity (MFI) of pHrodo™ fluorescent-positive cells to indicate the number of bacteria engulfed by each phagocytic cell (Figure 4).

To test the pHrodo™ dye-based system in bovine and buffalo species, a temperature-dependent negative control incubated at 4°C was used to inhibit the bacterial engulfment and phagosome–lysosome membrane fusion as showed by Neaga et al. (16). Predictably, the samples incubated on ice exhibited a low percentage of neutrophils and monocytes that engulf bacteria, as shown in Supplementary Figure S1.

The impact of species, temperature, time, and their interactions on the phagocytosis activity and capacity in bovine and buffalo phagocytes is presented in Table 3. For both species, significantly lower percentages and MFI values were observed for pHrodo^+^ neutrophils when the blood was incubated at 41°C compared with 39°C. Although a similar effect of temperature was observed for the pHrodo^+^ monocytes, the difference was significant in buffaloes only. Both temperature and heat exposure time showed significant effect on the phagocytosis activity of neutrophils (*p* ≤ 0.001) and monocytes (*p =* 0.004; *p* ≤ 0.001), while only phagocytosis capacity (MFI) of monocytes was affected by temperature (*p* = 0.001). A significant effect of species was observed on MFI of neutrophils (*p* = 0.035) and the percentage and MFI of monocytes (*p* ≤ 0.001) (Table 3).

**Table 3:** Comparison of the percentage values of pHrodo^+^ neutrophils and monocytes and their Mean Fluorescent Intensity (MFI) between bovine and buffalo after *in vitro* incubation at 39°C (normal temperature) and 41°C (hyperthermia).

	Bovine		Buffalo		*P*-value						
Temperature	39°C	41°C	39°C	41°C	Species	Temperature	Time	Species * temperature	Species * time	Temperature * time	Species * temperature * time
% pHRodo^+^ neutrophils	73.14 ± 4.04^**B**^	50.15 ± 4.85^**A**^	69.37 ± 2.81^**B**^	54.22 ± 3.86^**A**^	0.961	**≤0.001**	**≤0.001**	0.204	0.624	0.058	0.82
MFI neutrophils	63668.17 ± 11436.03^**C**^	27960.72 ± 3273.41^**A**^	67044.56 ± 5696.38^**C**^	53676.45 ± 4175.88^**B**^	**0.035**	**0.001**	0.204	0.103	0.616	0.781	0.451
% pHRodo^+^ monocytes	48.73 ± 3.34^**AB**^	44.02 ± 3.51^**A**^	70.72 ± 3.26^**C**^	56.17 ± 3.47^**B**^	**≤0.001**	**0.004**	**0.001**	0.127	0.71	0.376	0.649
MFI monocytes	37538.91 ± 2557.81^**A**^	33572.91 ± 2074.32^**A**^	90445.47 ± 12840.00^**B**^	70278.79 ± 8221.10^**B**^	**≤0.001**	0.153	0.222	0.335	0.15	0.636	0.867

Means with different capital superscripts (A, B, and C) differ significantly (P < 0.05) in column (39°C vs. 41°C and Bovine vs. Buffalo). Data are presented as means ± SE. Significant values are shown in bold.

**Figure 5:**
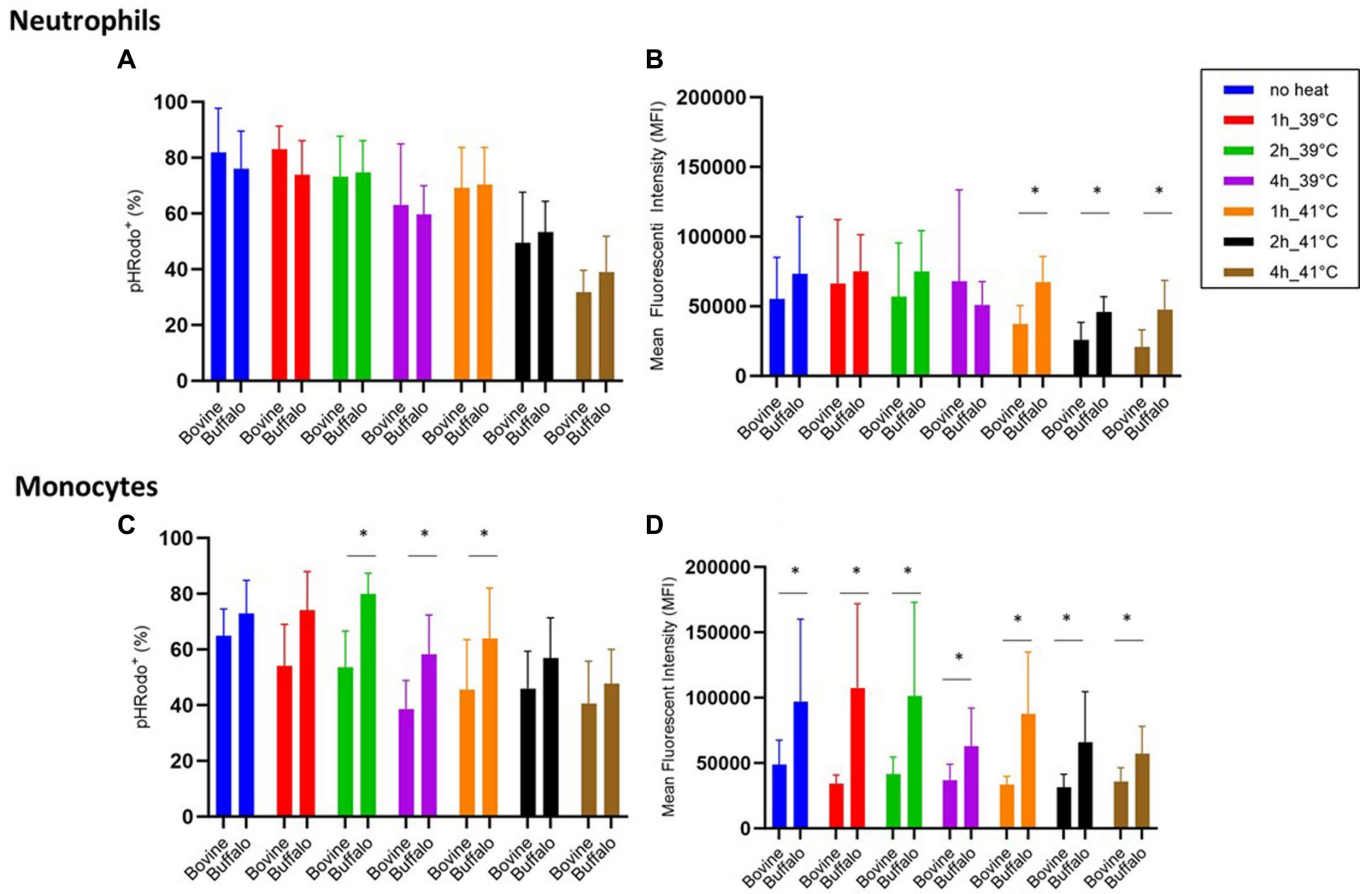
The impact of hyperthermia on bacterial phagocytosis by bovine and buffalo neutrophils and monocytes. The blood samples were incubated at different times and temperatures. For both species, no heat sample was used as control for basal level. The percentages of pHrodo^+^ cells and their MFI were calculated for gated neutrophils and monocytes. Data were presented as mean ± SEM (*indicates *p* < 0.05).

Although no significant differences were found between the two species regarding phagocytosis activity of neutrophils (Figure 5A), lower phagocytosis capacity values (MFI) were found in bovine cells compared with buffalo cells at 41°C (Figure 5B). Interestingly, buffalo monocytes showed significantly higher phagocytosis activity and capacity than bovines (Figures 5C,D).

The time course of phagocytosis activity and capacity of neutrophils and monocytes is presented in Table 4. Compared with samples with no heat treatment (fresh sample), blood samples from both species incubated for 4 h at 39 or 41°C showed decreased percentages of pHrodo^+^ neutrophils and monocytes. The values at 41°C were lower than values at 39°C only for neutrophils.

**Table 4:** Bovine and buffalo blood samples were incubated for different times as indicated, and the percentage of pHrodo™ fluorescent positive neutrophils and monocytes and the mean fluorescence intensity (MFI) of pHrodo™ fluorescent-positive cells were analyzed by flow cytometry using pHrodo™ Green *E. coli* Bioparticles^®^.

	% pHRodo + neutrophils		MFI neutrophils		% pHRodo + monocytes		MFI monocytes	
Bovine								
No heat	81.80 ± 6.45^b^	81.80 ± 6.45^c^	55249.68 ± 12210.24^a^	55249.68 ± 12210.24^c^	64.90 ± 3.90^b^	64.90 ± 3.90^b^	48685.57 ± 7691.25^b^	48685.57 ± 7691.25^b^
Temperature	**39°C**	**41°C**	**39°C**	**41°C**	**39°C**	**41°C**	**39°C**	**41°C**
1 h	83.15 ± 3.31^b**B**^	69.18 ± 5.94^c**A**^	66206.25 ± 18784.97^b**B**^	37184.37 ± 5494.718^b**A**^	54.02 ± 6.13^b^	45.58 ± 7.31^a^	34178.22 ± 2760.97^a^	33421.59 ± 2631.58^a^
2 h	73.24 ± 5.90^ab**B**^	49.51 ± 7.37^b**A**^	56845.22 ± 15724.49^a**B**^	25944.64 ± 5078.642^ab**A**^	53.51 ± 5.34^b^	45.95 ± 5.49^a^	41465.61 ± 5385.87^ab^	31604.00 ± 4026.65^a^
4 h	63.02 ± 8.96^a**B**^	31.75 ± 3.21^a**A**^	67953.05 ± 26824.99^b**B**^	20753.15 ± 5009.320^a**A**^	38.65 ± 4.17^a^	40.54 ± 6.20^a^	36972.90 ± 4969.45^ab^	35693.16 ± 4366.98^ab^
Buffalo								
No heat	76.00 ± 5.09^b^	76.00 ± 5.09^c^	73279.24 ± 15475.8^b^	73279.24 ± 15475.8^b^	72.8 ± 4.49^b^	72.8 ± 4.49^c^	97086.22 ± 23818.4^ab^	97086.22 ± 23818.4^b^
Temperature	**39°C**	**41°C**	**39°C**	**41°C**	**39°C**	**41°C**	**39°C**	**41°C**
1 h	73.85 ± 4.64^b^	70.39 ± 5.04^c^	74954.63 ± 9994.076^b^	67212.03 ± 7020.83^b^	74.00 ± 5.27^b^	63.94 ± 6.83^bc^	107420.62 ± 24347.51^b^	87764.82 ± 17823.16^ab^
2 h	74.59 ± 4.34^b**B**^	53.25 ± 4.20^b**A**^	75050.01 ± 11053.78^b**B**^	46023.57 ± 4137.01^a**A**^	79.97 ± 2.79^b^	56.91 ± 5.47^ab^	101241.01 ± 27142.32^ab^	65866.31 ± 14602.17^ab^
4 h	59.68 ± 3.88^a**B**^	39.01 ± 4.87^a**A**^	51129.04 ± 6252.487^a^	47793.75 ± 7868.24^a^	58.21 ± 5.34^a^	47.67 ± 4.66^a^	62674.75 ± 11097.90^a^	57205.24 ± 7892.11^a^

For both species, no heat sample was used as control for basal levels of phagocytosis. Data are presented as means ± SE. Means with different lowercases (a, b, and c) differ significantly (*P <* 0.05) in row (no heat vs. 1, 2, or 4 h); means with different capital superscripts (A, B, and C) differ significantly (*p <* 0.05) in column (39°C vs. 41°C).

## Discussion

The challenge of global warming and the resulting negative effects of heat stress on animal health and productivity reinforce the need for intensive research to identify animal species and breeds with greater thermal adaptation (17, 18). Profound impact of heat stress on the immune system has been reported for several animal species (19–22). Research on the immune system of water buffalo has recently gained special interest, leading to the characterization of key elements of the immune system with some similarities and differences from the bovine immune system (23–27). However, it is not yet known whether the two species show different thermotolerance capacities of their immune system. Therefore, the present study comparatively analyzed the impact of *in vitro* hyperthermia on cell viability and phagocytosis activity of leukocytes and their subpopulations in bovine and buffalo species.

The impact of heat stress on different physiologic functions of the body system has been described in several studies. This includes reduced production and reproduction capacities, a heat stress-associated anemia due to decreased erythrocyte numbers, decreased hemoglobin concentrations and reduced packed cell volume, and compromised innate and adaptive immune responses due to the direct effect of glucocorticoids and catecholamines on cytokine production, favoring polarization of the T-cell response toward a Th2-dominant response (28). The existence of species-specific thermal adaptation mechanisms has been intensively investigated in the literature. Responses of mammals to heat stress include physiological, behavioral, and metabolic responses such as increased water intake, sweating and respiration rates and reduced heart rate and feed intake (29). Humans and horses show higher capacity to dissipate heat, while other species such as dogs, cats, pigs, and buffaloes have few sweat glands, depending on other thermoregulation mechanisms (30).

The difference in thermoregulatory responses to heat stress in cattle and buffalo is primarily attributed to anatomical distinctions, such as epidermal color and thickness, hair density, and sweat gland density. Recent research has highlighted a more pronounced onset and severity of heat stress in buffaloes compared with cattle at various temperature-humidity index (THI) levels, suggesting potential variations in the timing and magnitude of the immune response to heat stress between the two species (31, 32). In this context, buffaloes exhibit a higher burden of oxidative stress, leading to a subsequent reduction in metabolic activity and immune response. Consequently, it is postulated that the impairment of different immune functions is more notable in heat-stressed buffaloes compared with cattle (31, 32).

The direct impact of hyperthermia on cell death has been demonstrated in several studies (33, 34). The mechanisms by which heat stress induces apoptosis involve the activation of a mitochondrial pathway, leading to generation of ROS, loss of mitochondrial membrane potential, release of cytochrome c from mitochondria, opening of permeability transition pores, expression of the Bcl-2 family members, and activation of caspases-9 and 3 (35). In addition, a mitochondria-independent apoptotic pathway has been described, involving the Ca^2+^−dependent cysteine protease calpain that induces caspase-12 localization on the cytoplasmic side of the ER (36). Detection methods of cell death include the analysis of membrane phosphatidylserine, changes in MMP, and caspase activity. In the present study, a fluorogenic substrate of caspases-3 and 7 was used in combination with a DNA-binding dye, to evaluate the impact of hyperthermia on cell viability. The advantage of this methodology relies on its high potential to differentiate between apoptotic and necrotic cells as activation of caspase-3 is a key marker of early apoptosis, while cell necrosis is associated with the lack of caspase activation and increased permeability of cell membrane (37).

For fresh leukocytes before incubation, lower cell viability with higher percentage of apoptotic cells was observed in bovine compared with buffalo cells, an effect that remained significant for several incubation times under hyperthermia. Whether this could be related to a higher natural resistance of buffalo cells to the hypotonic lysis during the cell separation procedure, it needs to be investigated in subsequent studies. For both species, lymphocytes showed higher sensitivity to *in vitro* hyperthermia compared with granulocytes and monocytes with increased apoptosis rates along with increasing incubation time. This seems in contrast to the higher heat shock resistance reported for camel lymphocytes compared to other subsets (13). In a previous study, we observed that bovine mononuclear cells were susceptible to prolonged standard *in vitro* cultivation by showing an increase in cells with active caspase-3 (38).

Here, the lack of significant changes in cell vitality reported for buffalo granulocytes under *in vitro* heat stress indicates higher thermotolerance of this cell population compared with their bovine counterparts, where 4 h of incubation at 41°C was associated with increased cell necrosis. This result contradicts the findings of Lecchi et al., who did not observe any impact of heat shock on vitality of bovine neutrophils (11). The different results could be related to the sample types used. In fact, purified neutrophils were used by Lecchi et al. (11) while whole blood was used here, which may imply a possible indirect effect of heat stress on neutrophils mediated by other leukocyte cell types. Another cause of different results could be due to the different assay used to evaluate the activity of caspases. Although long-time incubation (4 h) induced cell necrosis of monocytes from both species and under normal or hyperthermia temperatures, cell apoptosis was only observed in bovine monocytes under hyperthermia. The impact of hyperthermia on bovine monocytes seems to be in line with the previously reported decrease in cell viability with increased monocyte apoptosis after *in vitro* heat stress (10). Recent studies identified the heat shock protein 70 (HSP70) as key player in repairing cells and protecting from programed cell death during heat stress (39, 40). Therefore, future research could focus on the differential expression of HSP70 and its role in the different sensitivity to heat stress between bovine and buffalo cells.

Phagocytosis is a key function of innate immune cells, such as monocytes and neutrophils. The analysis of phagocytosis is usually evaluated using fluorochrome-labeled bacteria or bioparticles. A limitation of this method is the lack of distinction between ingested bacteria and those attached to the cell surface. To overcome this limitation, some studies used a fluorescent quencher to differentiate between attached and ingested bacteria. A recently developed pHrodo™ dye-based system has been validated for the assessment of actual phagocytic activity by measuring the acidification of bioparticles inside the phagolysosome because of their engulfment, thus eliminating the need for wash and quenching steps (16). The current study represents the first report validating the pHrodo™ methodology for the analysis of phagocytosis activity in bovines and water buffaloes and identifies the optimal incubation time and bioparticle/cell ratio in the two species. In the present study, comparative analysis of phagocytosis using the same protocol revealed an overall higher percentage of phagocytic cells (phagocytic activity) and the MFI of each cell (phagocytosis capacity) for buffalo monocytes compared with bovine monocytes. In neutrophils, this was observed for the phagocytosis capacity but not for the fraction of phagocytosis-positive cells. Being a receptor-mediated function, the efficacy of phagocytosis is influenced by the expression of specialized receptors that enhance the engulfment of target microbes through different mechanisms including the engagement with opsonizing antibodies or complement factors that cover the microbe. As the current study did not use opsonized bioparticles, a role for differential expression of phagocytic opsonin receptors, such as the FC receptors CD16, CD32, and CD64, and complement receptors (CR), such as CR3 (41, 42), in the observed differences cannot be hypothesized. However, a role of the differential expression of other phagocytosis receptors (43) such as scavenger receptors, C-type lectin receptors, and toll-like receptors in buffalo and bovine phagocytes could be the focus of further studies. In addition, given that classical monocytes (cM) are the main phagocyting monocyte subset in both bovine and buffalo, the enhanced phagocytosis activity of bubaline monocytes could be linked to the specific phenotype of bubaline cM being positive for the activation marker CD16^+^ (CD14^++^CD16^+^), which is in contrast to the CD16-phenotype (CD14^++^CD16^−^) of bovine cM (24).

Several previous *in vitro* and *in vivo* studies have reported reduced phagocytosis in bovine neutrophils under heat stress conditions (11, 44, 45). The results of the present study confirm the negative effect of heat stress on the phagocytosis function of bovine neutrophils and reveal the similar effect on buffalo neutrophils. This points to an impairment antimicrobial function of these phagocyte cells, which could be responsible for higher susceptibility of heat-stressed animals to microbial infections. Taking the time factor in account, the stress-induced effect on neutrophils started earlier in bovine (after 1 h) than buffalo (after 2 h) cells, indicating a relatively higher resistance of buffalo neutrophils to *in vitro* heat stress. In contrast, the fraction of phagocytosis-positive monocytes was reduced in both species at longer heat stress exposure time (4 h). However, this reduction could be due to the observed decrease in cell vitality. In fact, monocytes from both species did not significantly change in their phagocytosis function at any duration of *in vitro* heat stress. This result may indicate the existence of thermal adaptation mechanisms in monocytes rather than neutrophils, making the monocytes relatively more resistant to short heat stress.

One of the limitations of the current study is the low number of study animals. In addition, the observed effects of *in vitro* heat stress on the immune system need to be confirmed by the *ex vivo* analysis of the antimicrobial functions of phagocytes in heat-stressed bovine and buffalo animals.

## Conclusion

The current study represents the first report on the comparative analysis of the effect of *in vitro* heat stress on bovine and buffalo leukocyte populations. This study has revealed notable variations in the modulation of blood leukocyte viability and functions between bovines and buffaloes in response to different levels of hyperthermia, highlighting that buffalo cells exhibit relatively higher thermal adaptation compared with bovine cells. These findings not only have deepened our understanding of how heat stress affects immune responses in these two crucial species but have also pinpointed the specific degree and duration of hyperthermia exposure that compromise specific immune functions in each species. Further studies are required to explore the underlying mechanisms through which hyperthermia impacts the immune system and identify the gene candidates associated with higher thermal adaptation of the immune system of water buffalo that could be considerable utility for breeding studies.

## Data availability statement

The original contributions presented in the study are included in the article/Supplementary material, further inquiries can be directed to the corresponding authors.

## Ethics statement

The animal study was approved by the Institutional Ethics Committee of CREA-ZA. The study was conducted in accordance with the local legislation and institutional requirements.

## Author contributions

MS: Conceptualization, Data curation, Investigation, Methodology, Resources, Visualization, Writing – original draft. MA: Formal analysis, Software, Visualization, Writing – original draft, Writing – review & editing. FG: Writing – original draft, Writing – review & editing. AR: Writing – review & editing. MZ: Writing – review & editing. JH: Writing – original draft, Writing – review & editing. GM: Conceptualization, Data curation, Investigation, Methodology, Resources, Supervision, Visualization, Writing – original draft.

## Footnotes

^1^https://www.meteoproject.it/ftp/stazioni/monterotondo

## Supplementary material

Supplementary Figure 1 Histogram overlays of percentage of neutrophils and monocytes pHrodo^+^, representative of one bovine (above) and one buffalo (below). To test the pHrodo™ dye-based system a temperature-dependent negative control incubated at 4°C was used to inhibit the bacterial engulfment and phagosome-lysosome membrane fusion. Blood without pHrodo™ Green *E. coli* Bioparticles^®^ was used to set a marker for FL1 auto-fluorescence. In each histogram overlay four fresh sample with no heat treatment were compared: 2 samples incubated at 4°C without (4°C neg) and with pHrodo™ Green *E. coli* Bioparticles^®^ (4°C pHrodo), and 2 samples incubated at 39°C without (39°C neg) and with pHrodo™ Green *E. coli* Bioparticles (39°C pHrodo).
