# Supplementary figures and images for: Hyperthermia-induced changes in leukocyte survival and phagocytosis: a comparative study in bovine and buffalo leukocytes
Source: Front Vet Sci. 2024 Jan 23;10:1327148. doi: 10.3389/fvets.2023.1327148 (PMC10844375; doi:10.3389/fvets.2023.1327148)

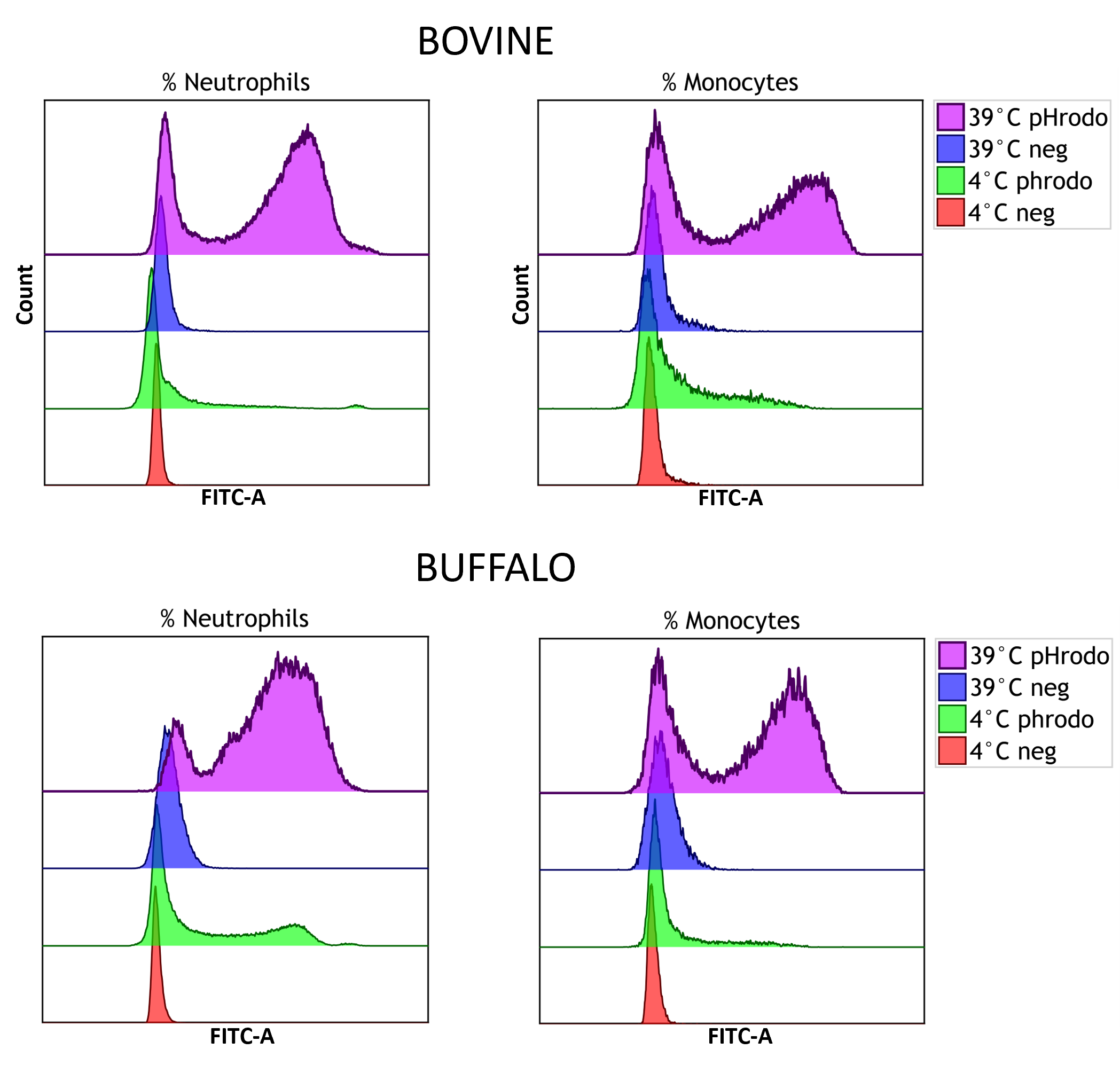

Supplement: Supplementary Figure 1 — Histogram overlays of percentage of neutrophils and monocytes pHrodo+, representative of one bovine (above) and one buffalo (below). To test the pHrodo™ dye-based system a temperature-dependent negative control incubated at 4°C was used to inhibit the bacterial engulfment and phagosome-lysosome membrane fusion. Blood without pHrodo™ Green E. coli Bioparticles® was used to set a marker for FL1 auto-fluorescence. In each histogram overlay four fresh sample with no heat treatment were compared: 2 samples incubated at 4°C without (4°C neg) and with pHrodo™ Green E. coli Bioparticles® (4°C pHrodo), and 2 samples incubated at 39°C without (39°C neg) and with pHrodo™ Green E. coli Bioparticles (39°C pHrodo). [file Image_1.TIF]
